# The Puzzling Coexistence of Eosinophilic Pneumonia With Sjogren’s Syndrome: A Diagnostic Dilemma

**DOI:** 10.7759/cureus.64470

**Published:** 2024-07-13

**Authors:** Jessica Liang, Mazhar Shapoo, Arabi Rasendrakumar

**Affiliations:** 1 Internal Medicine, Wayne State University Detroit Medical Center, Detroit, USA; 2 Rheumatology, Wayne State University Detroit Medical Center, Detroit, USA

**Keywords:** obstructive and restrictive lung diseases, anti-ssa, eosinophilia pneumonia, mepolizumab, rapidly progressive interstitial lung disease, idiopathic eosinophilic pneumonia, primary sjögren's syndrome

## Abstract

We present a case where a patient with no significant pulmonary nor autoimmune medical history presents with acute hypoxic respiratory failure and a dry cough that's made worse when conversing. She gets diagnosed with eosinophilic pneumonia after bronchoalveolar lavage (BAL) showed 70% eosinophils while also having labs highly suggestive of primary Sjogren's syndrome (pSS) with an anti-SSA titer of 111.3 U/mL and anti-SSA 52 kD Ab, immunoglobulin (Ig)G >200 U. The initial treatment plan was to start rituximab to target primary Sjogren's syndrome associated interstitial lung disease (pSS-ILD), however after close discussion with pulmonology, it was changed to mepolizumab to target eosinophilic pneumonia. From a diagnostic standpoint, it may be tricky to determine which disease process is driving the symptoms especially when the patient has labs that are convincing for both.

## Introduction

Primary Sjogren's syndrome (pSS) can lead to multi-systemic pathologies with interstitial lung disease (ILD) being a considerable contributor to mortality. When looking individually at the types of ILD, the prognosis of nonspecific interstitial pattern (NSIP), lymphocytic interstitial pattern (LIP), and organizing pneumonia (OP) are more favorable when compared to usual interstitial pattern (UIP) [1]. One retrospective study, which included 1,422 patients, with 44.9% of them having NSIP, showed that the mean survival time was nine years after confirmation of primary Sjogren's syndrome associated interstitial lung disease (pSS-ILD) [2]. One form of pneumonia that is rarely associated with pSS is eosinophilic pneumonia. We present a case where a patient with no significant pulmonary nor autoimmune medical history presents with acute hypoxic respiratory failure and gets diagnosed with eosinophilic pneumonia with labs highly suggestive of pSS.

## Case presentation

**Figure 1:**
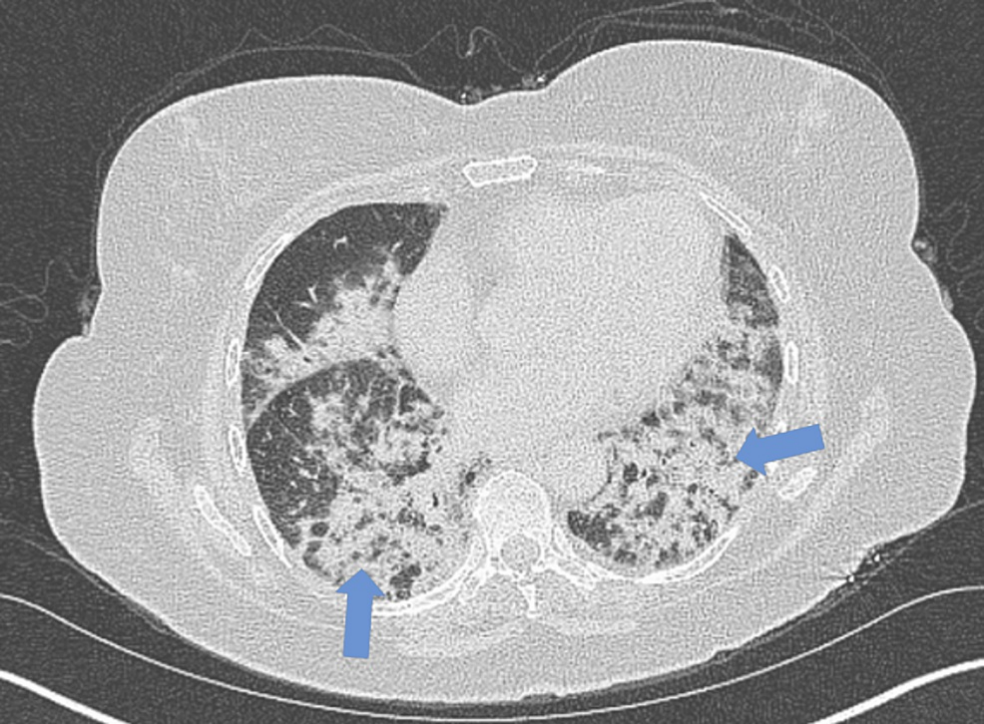
CT Thorax from outside the hospital upon initial symptom onset Extensive bilateral peribronchial consolidative changes, most prominent in the lower lobes, were noted (blue arrows).

A 76-year-old female with gastroesophageal reflux disease came to the hospital for a one-week history of dry cough (made worse when conversing) and shortness of breath upon ambulation. She was recently at an outside facility hospital a couple of weeks ago for the same symptoms. She does not have any smoking history, nor has she traveled recently. She works within the hospital and is not exposed to irritants that could cause occupational lung disease. She did not recall the specifics but stated that the autoimmune and infectious workups were negative at that time. Bronchoalveolar lavage (BAL) was not performed at that hospital. CT thorax showed severe bilateral lower lobe patchy infiltrate (Figure 1). They initially started her on treatment for community-acquired pneumonia with methylprednisolone, ceftriaxone, and azithromycin. Ultimately, they diagnosed her with cryptogenic organizing pneumonia, and she was discharged with a 10-day course of prednisone 50 mg and home oxygen as needed. She stated the steroids helped her breathe, but the exertional dyspnea reoccurred once she finished her course of steroids. She brought herself to the hospital once she started requiring a 4-5 L nasal cannula at home.

The results of laboratory tests performed at the time of admission are described in Tables 1, 2.

**Table 1:** pSS-related markers upon admission ANA: Antinuclear antibodies; Ig: Immunoglobulin; pSS: Primary Sjogren's syndrome associated interstitial lung disease; Anti-SSA: Anti-Sjogren's Syndrome A; Anti-SSB: Anti-Sjogren's Syndrome B; Anti-SSA 52 kD Ab, IgG:  Anti-Sjogren's Syndrome A 52 kD Antibody, immunoglobulin G; Anti SCL 70: Anti scleroderma 70 kD; Anti U1 RNP: Anti-U1-ribonucleoprotein; U3 RNP: U3 ribonucleoprotein.

PSS-Related Markers	Result
ANA	1:160
Anti-SSA	111.3 U/mL
Anti-SSB	3.2 U/mL
Anti-SSA 52 kD Ab, IgG	>200 Units
Anti SCL 70	2.7 U/mL
Anti-U1-RNP	<20 Units
U3 RNP	NEGATIVE

**Table 2:** General immunologic and infectious markers upon admission CRP: C-Reactive protein; PCR: Polymerase chain reaction; C3: Complement C3; C4: Complement C4; RF: Rheumatoid factor; ANCA IFA: Antineutrophil cytoplasmic antibodies; Anti-Jo1: Anti-histidyl tRNA synthetase; Anti-RNP: Antinuclear ribonucleoprotein; HIV: Human immunodeficiency virus.

General Markers	Result
CRP	134.4 mg/L
C3	108 mg/dL
C4	22 mg/dL
RF	12 IU/mL
ANCA IFA	Not detected
ANTI-JO1	0.7 Units
ANTI-RNP	1.7 Units
Mycoplasma PCR	Negative
HIV Antibody/Antigen Test	Negative

A high dose of methylprednisolone 125 mg every six hours was started given the acute increase in oxygen demand and extensive involvement of the lower lobes on CT. Given her significantly elevated anti-SSA, rheumatology was consulted for possible pSS-ILD. Bedside Schirmer's test was performed and it was weakly positive in the left eye. Interestingly, the patient denied any sicca symptoms. Even though she met the classification criteria for pSS, she did not meet the inclusion criteria since she was asymptomatic. Five days later, she continued to desaturate to the 70s when ambulating with no improvement in oxygen demand, which prompted the decision to treat her for pSS-ILD with rituximab and to perform a BAL. BAL was done on day seven of admission and cell differential showed 70% eosinophils (Table 3). This raised suspicion for eosinophilic pneumonia. Since she has been on steroids from previous hospitalization and the current admission, peripheral blood has been showing an absolute eosinophil count of 0. The prior year, she had a low absolute eosinophil count of 0.2. Eventually, after being on methylprednisolone 125 mg every six hours for seven days, steroids were tapered to prednisone 60 mg daily oxygen.

**Table 3:** Cell count differential from BAL BAL: Bronchoalveolar lavage

Cell Type	Percentage
RBC	34/mm^3^
Nucleated cells	21 /mm^3^
Neutrophils %	2
Lymphocytes %	2
Monocytes %	3
Eosinophils %	70
Basophils %	5
Macrophages %	14
Cells Counted	100

**Figure 2:**
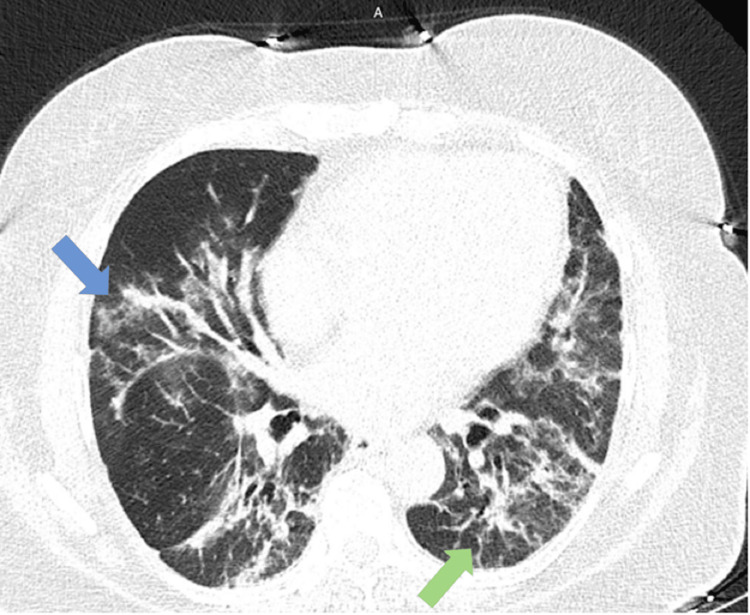
High-resolution CT thorax High-resolution CT (HRCT) after methylprednisolone 125 mg every 6 hours x 7 days and prednisone 60 mg x 4 days. Ground glass opacities (blue arrow) and reticular opacities (green noted) were noted. Features are consistent with cryptogenic organizing pneumonia or nonspecific interstitial pneumonia.

A high-resolution CT thorax was performed and it showed only very mild improvement in the ground glass and reticulonodular opacities (Figure 2). Oxygen requirement slowly improved by day 12 of admission. She was discharged with home oxygen with the following long steroid taper: prednisone 30 mg daily for 14 days then 20 mg for 30 days.

Two weeks post-discharge, the patient began to show side effects from the prednisone with a hemoglobin A1c of 7.0%, muscle atrophy of the left lower extremity, and skin hyperpigmentation in the left shin. An outpatient pulmonary function test (PFT) was done and the results confirmed a restrictive process with significantly decreased lung function forced vital capacity (FVC) of 1.33L along with decreased diffusing capacity of the lung for carbon monoxide (DLCO) and total lung capacity (TLC). There was no response to bronchodilation, which confirms she does not have asthma. She was able to walk 70% of the predicted distance on a 6-minute walk test. A radioallergosorbent test (RAST) panel was ordered and she tested positive for multiple allergens such as cockroach IgE, common ragweed IgE, dust mite IgE, and others. In the setting of a positive RAST panel and eosinophilic pneumonia, side effects from prednisone, and the patient's age, it was decided to pursue treatment with mepolizumab. 

One and half months post-discharge, the patient continued to demonstrate the side effects of prolonged steroid taper with cushinoid faces. A decision was made to decrease steroid intake to 20 mg every other day and to start methotrexate 10 mg weekly as steroid-sparing therapy. Shortly after this clinic visit, mepolizumab 100 mg every four weeks was approved by insurance. 

**Figure 3:**
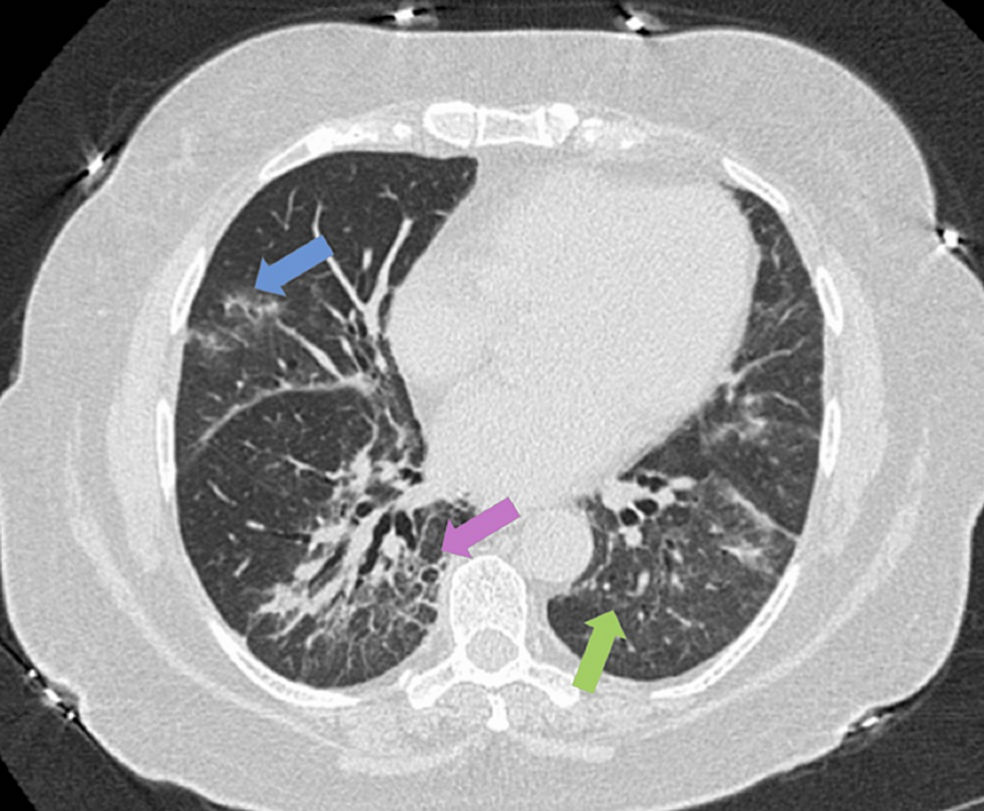
HRCT after two rounds of mepolizumab High-resolution CT thorax (HRCT) after two rounds of mepolizumab administration. Ground glass opacities (blue arrow) and reticular opacities (green arrow) appear mildly improved compared to the previous; however, bronchiectatic changes were noted in lower lobes (purple arrow).

Three months after discharge, she received two rounds of mepolizumab while continuing methotrexate and steroids. Repeat CT showed noticeable improvement in the bilateral ground glass and reticulonodular opacities in the lower lobes (Figure 3). A 6-minute walk test improved the walking by 77% of the predicted distance.

## Discussion

According to the European Alliance of Associations for Rheumatology/American College of Rheumatology (EULAR/ACR) criteria for pSS, sicca symptoms are required for the diagnosis to satisfy the inclusion criteria. Sicca symptoms are usually thought of as xerostomia and dry eyes; however, variations in the sicca symptoms have been reported. Oral dryness can manifest as soreness, adherence of food to buccal surfaces, fissuring of the tongue, and dysphagia [3]. It is worth considering that the patient's Gastroesophageal Reflux Disease (GERD) symptoms may be a variation of xerostomia as decreased saliva leads to increased acid in the abdomen. On the other hand, if GERD is not a manifestation of xerostomia, it is also not uncommon for pSS to not initially present with sicca symptoms. In a case-control study studying 102 pSS patients, pulmonary manifestation was actually the second most common initial manifestation of pSS (n = 16, 15.7%) [4]. It also found that 52% of pSS-ILD presented with non-sicca onset including pulmonary symptoms (15.7%) and joint involvement (10.88%). Pulmonary complications were more progressive and severe in non-sicca onset pSS-ILD when compared to sicca onset patients. This correlates with the patient's acute hypoxic respiratory failure and extensivity of pulmonary involvement since she also did not present with overt sicca symptoms. 

BAL is not routinely performed to diagnose pSS-ILD but rather used to exclude other parenchymal diseases. BAL more commonly shows lymphocytosis in pSS. Interestingly, our patient's BAL was significantly high in the eosinophilic cell count. The criteria for acute eosinophilic pneumonia is eosinophils >25% in BAL. Common associations of acute eosinophilic pneumonia are chronic myelogenous leukemia (CML), HIV infection, and smoking whereas chronic eosinophilic pneumonia is associated with asthma [5]. The patient did not have any of these medical and social histories. The most convincing abnormalities in her workup were her high anti-SSA and Anti-SS-A 52kD IgG titers, which makes one highly consider if this could be an atypical case of pSS-ILD before dismissing an autoimmune component to her clinical course.

## Conclusions

The association between Sjogren's syndrome and eosinophilic pneumonia is uncommon. From a diagnostic standpoint, it may be tricky to determine which disease process is driving the symptoms when labs are convincing for both. We directed the treatment towards eosinophilic pneumonia since the patient did not entirely meet the diagnostic criteria for pSS yet had convincing lab results for eosinophilic pneumonia. Ultimately, treatment modality should be determined via close discussion between pulmonology and rheumatology and more research is needed to study the possible link between pSS and eosinophilic pneumonia.

## References

[1] Enomoto Y, Takemura T, Hagiwara E (2013). Prognostic factors in interstitial lung disease associated with primary Sjögren's syndrome: a retrospective analysis of 33 pathologically-proven cases. PLoS One.

[2] Gao H, Sun Y, Zhang XY (2021). Characteristics and mortality in primary Sjögren syndrome-related interstitial lung disease. Medicine (Baltimore).

[3] Kassan SS, Moutsopoulos HM (2004). Clinical manifestations and early diagnosis of Sjögren syndrome. Arch Intern Med.

[4] Gao H, Zou YD, Zhang XW, He J, Zhang J, Sun Y, Li ZG (2018). Interstitial lung disease in non-sicca onset primary Sjögren's syndrome: a large-scale case-control study. Int J Rheum Dis.

[5] Pahal P, Penmetsa GK, Modi P, Sharma S (2024). Eosinophilic pneumonia. https://www.ncbi.nlm.nih.gov/books/NBK537169/.

